# Liver injury caused by fenofibrate within 48 h after first administration: a case report

**DOI:** 10.1186/s12876-021-01874-7

**Published:** 2021-07-29

**Authors:** Yu He, Ming-zhao Qin, Yi-wen Chen

**Affiliations:** Department of Geriatrics, Beijing Tongren Hospital, Capital Medical University, 1 Dongjiaominxiang street, Dongcheng District, Beijing, 100730 People’s Republic of China

**Keywords:** Fenofibrate, Liver injury, Within 48 h, Case report

## Abstract

**Background:**

Fenofibrate is commonly used in the treatment of dyslipidemia. Fenofibrate is related to mild aminotransferase elevations and in some cases severe chronic injury such as fibrosis or cirrhosis, resulting in liver transplantation or death. The latency of disease has been reported to range between weeks to years.

**Case presentation:**

A 63 years old male with hypertriglyceridemia developed symptoms of fatigue and anorexia 48 h after taking fenofibrate for the first time. The patient’s aminotransferase level was more than 10 times ULN. Immediately, fenofibrate was discontinued and aminotransferase level returned to normal 23 days later. To assess causality between the drug and liver damage, the standardized Roussel Uclaf Causality Assessment Method (RUCAM) was used. The patient's RUCAM score was 7, which fell in the group of “probable”. Eight months later, follow-up examination suggested the liver function was normal.

**Conclusions:**

Weakness, fatigue and abnormal liver function during fenofibrate therapy should be closely monitored and trigger prompt withdrawal if these symptoms occur.

## Background

Fenofibrate is a peroxisome proliferator receptor alpha (PPAR-α) activator which is commonly used in the therapy of hypertriglyceridemia and mixed dyslipidemia. Mild, transient serum aminotransferase elevations may develop in up to 20% of patients receiving fenofibrate. Only 3–5% of patients would exhibit elevation to above 3 times of ULN. In some cases, patients may develop severe chronic injury such as fibrosis or cirrhosis, which could lead to liver transplantation or death [1]. It is widely recognized that liver damage usually occurs several weeks or months after starting of the medication [2], with some cases extending to 6 months or even years after starting of the medication [3].

## Case presentation

A 63 years old male with hypertriglyceridemia [triglyceride (TG) 12.82 mmol/L], started taking fenofibrate 200 mg/day for the first time. One day before taking fenofibrate, liver function tests were performed. The laboratory result showed alanine aminotransferase (ALT), aspartate aminotransferase (AST), alkaline phosphatase (Alk P) were all in normal range. After taking fenofibrate for 2 days, the patient developed fatigue, body aches, anorexia and dark urine, fenofibrate was discontinued immediately. There was no change to the rest of the patient’s medication. One day after withdrawal, liver function tests showed ALT 690 U/L, AST 521 U/L, the direct bilirubin (DBIL) 50.1 umol/L, the total bilirubin (TBIL) 75.8 umol/L, Alk P 202 U/L, γ-GGT 769 U/L, the International Normalized Ratio (INR) 0.93.

**Fig. 1:**
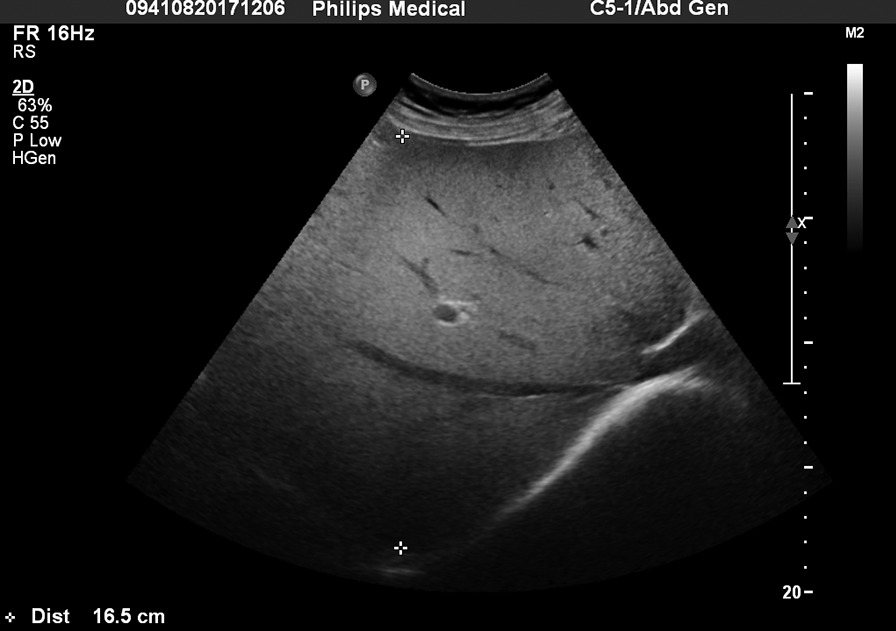
Abdominal ultrasound showed an enlarged, fatty liver with enhanced liver parenchymal echogenicity

Patient’s body mass index (BMI) was 27.76 kg/m^2^ with a body surface area (BSA) of 2.21 m^2^. He was jaundiced with yellow sclera and skin. He presented with right upper quadrant tenderness and hepatic percussion pain was elicited. Hepatitis B surface antigen and hepatitis C antibody were negative. Anti-nuclear antibodies, anti-smooth muscle antibodies, anti-liver and kidney microsome antibodies, anti-mitochondrial antibodies, anti-myocardial antibodies, anti-parietal cell antibodies, anti-neutrophil cytoplasmic antibodies, and anti-ENA antibody were all negative. The IgG was normal (933.31 mg/dL), and the IgM was slightly decreased (36.45 mg/dL). Abdominal ultrasound showed an enlarged, fatty liver with enhanced liver parenchymal echogenicity (Fig. 1). Additionally the US showed a normal gallbladder size, normal gallbladder wall thickness, and multiple gallstones.

Six days after withdrawal bilirubin levels decreased to normal. Ten days after withdrawal, AST and ALP level decreased to normal, fatigue and anorexia was relieved. The relationship between liver function and fenofibrate is shown in the Table 1.

**Table 1:**
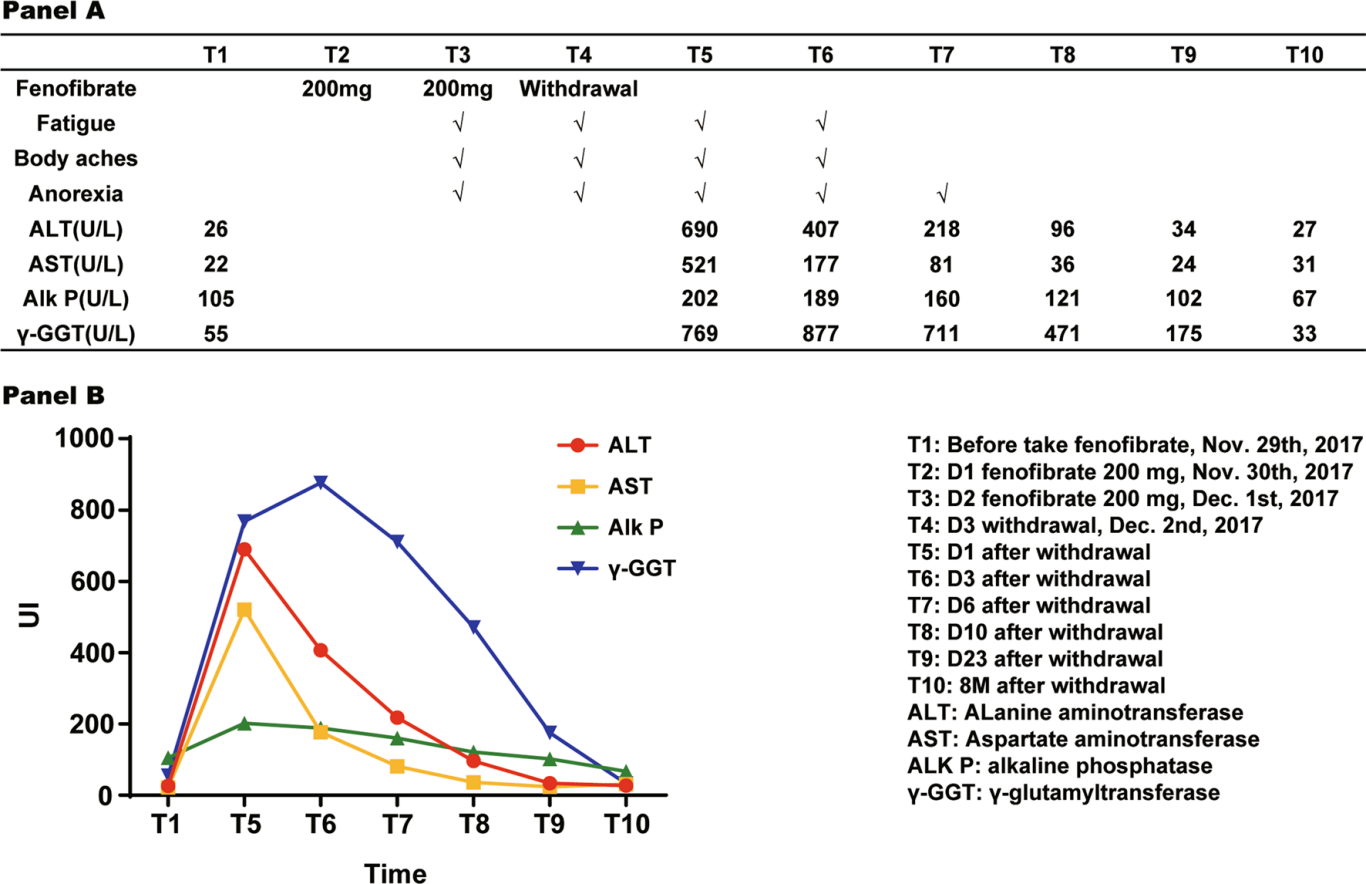
The relationship between liver function and fenofibrate

All evidences suggested that this patient’s liver injury were attributed to fenofibrate. Although repeated application of fenofibrate on the patient would have confirmed its pathogenicity, fenofibrate was not re-applied because of the severity of liver damage.

To assess causality between the drug and liver damage, the standardized Roussel Uclaf Causality Assessment Method (RUCAM) [4] was used. The patient's RUCAM score was 7, which fell in the group of “probable”. To categorize the pattern of liver damage the R-ratio was calculated [5]: [ALT/Upper Limit of Normal (ULN)] ÷ [Alk P/ULN]. The patient’s R-ratio was 12.8 and ALT ≥ 3 ULN, which fitted hepatocellular damage type.

## Discussion and conclusion

Recent literature reported that among 1229 patients with confirmed drug induced liver injury (DILI), 7 cases (0.6%) were attributed to fenofibrate. The latency was short (5–8 weeks) in 4 patients but much longer (18–56 weeks) among the rest [1]. Previously, it was reported that a 53 year old female developed severe abdominal pain 36 h after re-application of fenofibrate [6]. But the uniqueness of the very patient mentioned in this article is that he was a first-time fenofibrate user, and liver damage occurred within 2 days of taking the drug.

This patient was not examined for hepatitis A IgM antibody and hepatitis E IgM antibody as he was living in Beijing, which was not the epidemic area of the above mentioned diseases. His aminotransferase level was normal before commencing fenofibrate. Aminotransferase levels returned to normal 23 days after withdrawal, which was also not in accordance with the course of hepatitis A and E as well.

Patient’s past medical history included dyslipidemia, recurrent chylemia, hypertension, hyperuricemia, Type 2 diabetes, sleep apnea hypopnea syndrome and neurodermatitis. He took telmisartan, felodipine, arotinolol hydrochloride, allopurinol, and metformin as usual medications. These drugs rarely induce liver damage.

The mechanism of hepatotoxicity of fenofibrate is not known but appears to be immunological [7] In case the patient visits again, low-frequency HLA type HLA-A*33:01 shall be tested, which is associated with DILI [8].

The symptoms of liver injury occurring 48 h after taking fenofibrate, suggests that liver injury may have occurred promptly after the initial use. Examination of liver function should be taken before taking medicine. Any symptom of fatigue, poor appetite and yellow stained skin and sclera during fenofibrate therapy should trigger prompt withdrawal. Severe chronic injury and mortality may occur if drug discontinuation is delayed.

## Data Availability

All data generated or analyzed during this study are included in this published article.

## Abbreviations

PPAR-α: peroxisome proliferator receptor alpha
TG: triglyceride
ALT: alanine aminotransferase
AST: aspartate aminotransferase
AlkP: alkaline phosphatase
γ-GGT: γ-glutamyltransferase
DBIL: direct bilirubin
TBIL: total bilirubin
INR: the International Normalized Ratio
BMI: body mass index
BSA: body surface area
DILI: drug induced liver injury
